# Mindful Eating: Connecting With the Wise Self, the Spiritual Self

**DOI:** 10.3389/fpsyg.2018.01271

**Published:** 2018-08-14

**Authors:** Jean L. Kristeller, Kevin D. Jordan

**Affiliations:** Department of Psychology, Indiana State University, Terre Haute, IN, United States

**Keywords:** mindfulness, mindful eating, spirituality, wisdom, binge eating, obesity

## Abstract

In the Mindfulness-Based Eating Awareness Training program (MB-EAT) (Kristeller and Wolever, 2014; Kristeller and Wolever, in press), mindfulness practice is taught, mindful eating is cultivated, and self-acceptance and spiritual well-being are enhanced. An integrative concept is the value of cultivating ‘wisdom’ in regard to creating a new and sustainable relationship to eating and food. ‘Wisdom’ refers to drawing on personal experience and understanding in a flexible, insightful manner, rather than strictly following external rules and guidelines. Several clinical trials involving variations of MB-EAT have documented substantive improvement in how people relate to their eating, including individuals with both binge eating disorder (BED) and subclinical eating issues. Based on the traditional value of contemplative practices for cultivating spiritual engagement, and on evidence from related research showing that spiritual well-being increases in the Mindfulness-Based Stress Reduction (MBSR) program and is related to other effects, we hypothesized that the MB-EAT program would also engage this aspect of experience, as assessed by the Functional Assessment of Chronic Illness Therapy – Spiritual Well-Being subscale (FACIT-Sp), and that increases in spiritual well-being would relate to other measures of adjustment such as emotional balance and improvement in disordered eating. Participants (*N* = 117) with moderate to morbid obesity, including 25.6% with BED, were randomly assigned to MB-EAT or a wait-list control, and assessed on the FACIT-Sp and other measures at baseline, immediate post (IP), and 2-month followup (F/Up). Both FACIT-Sp factors [Meaning/Peace (M/P) and Faith] increased significantly in the MB-EAT group and were stable/decreased in the control group. Increases in these factors related to improvement in emotional adjustment and eating regulation at IP and at F/Up, and to increases in aspects of mindfulness measured by the Five Facet Mindfulness Questionnaire (FFMQ). Increases in M/P during treatment mediated effects of the FFMQ Observe factor on eating regulation and depression at IP. Results are discussed in terms of the role that mindfulness practice plays in cultivating ‘wise mind’ and the related value of spirituality. It is argued that the core elements of the MB-EAT program lead to meaningful spiritual engagement, which plays a role in people’s ability to improve and maintain overall self-regulation.

## Introduction

Mindfulness-Based Eating Awareness Training (MB-EAT) (Kristeller and Wolever, 2014; Kristeller, 2016; Kristeller and Wolever, in press) integrates the science of meditation practice with the science of our relationship to eating, nutrition, food and body awareness, coupled with theories of self-regulation (Carver and Scheier, 1998; Shapiro and Schwartz, 2000). Clinical trials have shown significant improvement in eating regulation, both in those with and without binge eating disorder (BED), and improvement in depression (Kristeller and Hallett, 1999; Kristeller and Wolever, 2011; Kristeller et al., 2013; Kristeller et al., in review). The core program has evolved over several decades, incorporating a wide range of mindfulness meditation practices, eating awareness practices, and guided practices linked to self-awareness and self-acceptance, and been adapted to a range of populations, including individuals with varying levels of obesity, with and without BED, and Type II diabetes (Miller et al., 2012; Daubenmier et al., 2016; Mason et al., 2016).

Throughout the MB-EAT-based programs, regardless of variations, an integrative concept is the value of connecting to the ‘wise self’ in the service of self-regulation, as an alternative to drawing on externally imposed rules of dieting, food restriction, and socially imposed norms regarding body weight and appearance. What do we mean by the ‘wise self’ and how might this be connected to our ‘spiritual’ self? Although meditative and spiritual traditions are closely linked, this aspect of meditation-based practice, particularly within therapeutic contexts, has received relatively little systematic attention within contemporary research and theory. In some ways this is easily understood. Mindfulness practice is commonly – and usefully – framed as a cognitive process, in which attention is trained to rest gently on the breath or body experience, and then expanded further onto thoughts or particular emotional experiences, without being drawn into reactive or judgmental conditioned responses. The intent, drawn from traditional Buddhist theory, is to release the mind from highly conditioned reactivity or suffering, both in the moment and in more extended ways. Such effects have been clearly documented in areas of reduced stress reactivity, and improvement in behavioral and emotional self-regulation (Wachholtz and Pargament, 2005). The contemporary concept of ‘wisdom’ is also usefully drawn upon; it will be argued that this term implies greater access to parts of the brain that can improve self-regulation and well-being when presented with complex choices, as is true in many aspects of daily life, but that wisdom does not necessarily entail spiritual engagement, as it extends into all areas of functioning (Sternberg, 1990, 1998; Schwartz and Sharpe, 2010).

A growing area of research over the last 20 years has demonstrated the importance of spiritual engagement, as a psychological construct distinct from religious involvement (Hill et al., 2000; Koenig L.B., 2015), in many different populations including emerging adults, individuals with chronic illnesses, psychiatric patients, and older adults (Koenig H.G., 2015; McCarthy et al., 2015; Akin and Akin, 2017; Da Silva and Pereira, 2017; Chaar et al., 2018). The construct of spirituality has been examined in a multitude of ways including meaning in life (Bloch et al., 2017), purpose, spiritual coping (Charzyńska, 2015), compassion, and spiritual experiences (Knabb and Vazquez, 2018). These studies reflect a growing accumulation of research that suggests spirituality is associated with improved mental health, quality of life, and adjustment.

While the expression of spiritual experiences may be varied, the capacity for spiritual engagement may be more easily measured by assessing the components of spirituality such as meaning, peace, an experienced connection with religious faith/beliefs, and connection with something sacred/universal. The authors of the present article believe that this capacity for spiritual engagement is virtually universally reflected in many peoples’ desire to “step back” from their strivings and efforts to experience a more content and spiritual life. While often overlapping with religious engagement, it is understood to refer to underlying psychological experience, not inherently linked into culturally specific (or personally significant) sets of beliefs (Atchley, 2009).

Individual differences in spirituality are assessed through a variety of self-report measures, with one of the important distinctions among measures being the degree to which specific beliefs are still referred to, or incorporated into the measure (such as belief in God). One of the most prominent measures, the Functional Assessment of Chronic Illness Therapy – Spiritual Well-Being subscale (FACIT-Sp), assesses two factors, Meaning and Peace, and Faith (Peterman et al., 2002). It was developed to be applicable across individuals from different faiths, including atheists, demonstrates good psychometric properties, and is frequently used in the health literature. Cross-sectional studies suggest that meaning, peace, and faith beliefs as measured by the FACIT-Sp are associated with quality of life in cancer patients as well as self-reported emotional equanimity, better cognitive functioning, and social support (Kristeller et al., 2011; Jordan et al., 2014; Chaar et al., 2018). Interventional studies using a variety of mindfulness-based treatments have led to improvements in health-related quality of life (Gross et al., 2017), reduced chronic pain-related stress (Feuille and Pargament, 2015), and spirituality as assessed by the FACIT-Sp (Fish et al., 2014). Use of it to measure spiritual well-being in the MBSR program demonstrated significant increases on both factors, correlated with increases on measures of mindfulness and improvement on other measures of well-being, including depression, anxiety and reported medical symptoms (Carmody et al., 2008).

To the extent that mindfulness can lead to spiritual engagement (Kristeller and Jordan, 2018), it is expected that health benefits likely accrue in part due to improved psycho-biological regulation (for review, see Hulett and Armer, 2016). These improvements may also interact with improved behaviors that are associated with health. For example, prayerful states and meditation are associated with activation in the prefrontal cortex (Newberg and d’Aquili, 2001; Azari et al., 2005; Seybold, 2007; Goleman and Davidson, 2017), an area of the brain that plays a role in self-regulation. In much the same way as a muscle, “working out” one’s self-regulation muscle may provide it with greater strength and ability to apply it in other ways and settings (Baumeister et al., 2006). Meditation training has been shown to improve self-regulatory processes and outcomes, lower cortisol responses, and increase secretory immunoglobulin A responses (Tang et al., 2007).

Exploring these processes within the arena of eating behavior may seem like an unlikely context. After all, food choice is about survival, basic health, pleasure, neither over-eating nor under-eating, but not necessarily about engaging any level of higher meaning or experience. The ways that mindfulness helps focus awareness on eating experience, thoughts, emotions, body acceptance, and managing stress reactivity should well suffice for understanding the increasingly substantial evidence regarding the value of mindfulness-based eating programs (Godfrey et al., 2015).

Eating-related practices are carefully informed by research on food intake regulation, such as heightening awareness of physiological vs. environmental or emotional ‘hunger’ triggers (Polivy and Herman, 1999; Wansink et al., 2005; Crespi and Unkefer, 2014); the role of sensory-specific satiety (referred to in the program as taste satisfaction/‘taste-satiety’) in modulating food intake (Guinard and Brun, 1998); awareness of stomach fullness to signal the end of a meal (Geliebter and Hashim, 2001; Herbert et al., 2013); and the natural variability and flexibility of eating patterns observed in ‘balanced’ eaters (Kristeller and Rodin, 1989). The program emphasizes eating for ‘quality over quantity,’ or deriving enjoyment from the moment-to-moment experience of eating, rather than from the amount of food ingested, thereby shifting the reward value of food without removing it (Stice et al., 2009).

Yet the broader intention for MB-EAT is helping individuals to let go of over-preoccupation with issues related to eating and weight, shifting their focus and energy back onto higher level concerns and values in their lives. The program draws on the perspective that all aspects of human functioning can be heightened through the use of mindfulness practice, and that these include spiritual capacity as part of cultivating wisdom (Young-Eisendrath and Miller, 2000; Kristeller, 2003; Jeste et al., 2010). MB-EAT draws on the concept of ‘wisdom’ throughout the program. From the first session, the concept of wisdom is introduced to underscore the value of mindfulness practice to look inside oneself for how to make choices about what to eat, when to eat, how much to eat, rather than depending on the strict rules of the dieting mentality. The use of the term ‘wisdom’ seems to resonate strongly with program participants, as does the program’s emphasis on flexibility, letting go of self-judgment and searching in a flexible way to develop new patterns of eating and food choice. (Kristeller, 2003, 2007).

We emphasize that ‘wiser’ alternatives may become apparent, even in the middle of eating, by gently bringing into one’s awareness new alternatives. As an example, a woman in our program confessed that she often ate donuts that had been brought into her office’s breakroom guiltily and compulsively. She came to Session 5, excitedly sharing that someone had brought in a box of donuts that week, and that she’d responded quite differently. She’d first checked that she was actually physically hungry, mindfully chose a donut that particularly appealed to her, and brought it back to her office to allow herself to fully attend to the experience. She noted that the first bite was exceptional (with no flavoring of guilt), the second bite was almost as good – but that the third bite was too sweet, too greasy, and the chocolate icing had lost its flavor. She paused, breathed – and threw out the rest of donut. She exclaimed to the group that she had never thrown out partially eaten food before in her life. This is an example of connecting with the ‘wiser’ self.

The MB-EAT program (Kristeller and Hallett, 1999; Kristeller and Wolever, 2011; Kristeller, 2016; Kristeller and Wolever, in press) incorporates a wide range of mindfulness-based practice, including sitting meditation practice; ‘mini-meditations’ lasting from a few moments to a few minutes, to be used while eating and at other times; guided eating practices, beginning with four raisins, and then incorporating increasingly challenging foods and food situations; guided meditations; and substantial home practice and group discussion. Again, the term ‘wisdom’ is used in many of the guided meditations, culminating in a Wisdom Meditation in Session 10. It is not until this practice that the word ‘spirituality’ is explicitly mentioned, yet it is not uncommon that participants, most of whom identify as Christian, note that their meditation practice sometimes evokes the tone of prayer, and that they have had multiple spiritual experiences as part of their meditation experience.

This leads back to the original question. How might our concepts of ‘wisdom’ and ‘spirituality’ be understood, both experientially and within the neuro-science of functioning, as related to cultivating capacities linked to mindfulness practice? These terms are often linked in the literature on the value of religious and spiritual practices, with an appreciation that these traditions help individuals not only to experience their own spirituality more deeply, but also help them to engage wisdom and greater well-being for themselves and in their communities (Young-Eisendrath and Miller, 2000; Henry, 2013). Given these strong historical linkages, it may be that meditation practice, in loosening the domination of highly conditioned reactive patterns related to survival behaviors (such as eating), and emotional responses that may be largely seated in the lower brain regions, inherently opens up access to those parts of the mind involved in spiritual experience. And it then may be that these spiritual experiences engaged during meditative practice ‘feed back’ to modulate the power of these more primitive linkages by facilitating access to more complex choices, or ‘wisdom,’ heightening overall self-regulation (Kristeller, 2007; Sharp, 2011).

Therefore, the specific aims of this paper were to explore whether earlier results regarding the impact of the MBSR program on spiritual well-being (also measured by the FACIT-Sp) (Carmody et al., 2008) would extend to a more focused mindfulness-based program, in regard to emotional and eating-related outcomes, and to explore the role of changes in mindfulness, as measured by the FFMQ, on such effects. Note: the primary results of the current randomized clinical trial are reported elsewhere (Kristeller et al., in review).

## Materials and Methods

### Participants

Sixty-one participants were randomly assigned to the MB-EAT group and 56 participants to the wait list control group. Completers were considered those who participated at least seven of the 10 weekly sessions. Of the study sample, 87% were female, and the race/ethnicity makeup was 88% white, non-Hispanic, 9% African–American, and 3% other. The mean age was 49.9 years (*SD* = 12.3, range = 19–76). Average BMI was 42.5 (range = 35–63.8). All were recruited in Terre Haute, IN, United States. All study procedures were approved by the ISU Institutional Review Board and all participants provided informed consent. Participants were solicited through local advertisements that mentioned a “new” approach to weight loss for individuals who were at least 50 lbs. overweight, but did not mention mindfulness to decrease bias. Individuals were screened in who had a BMI of 35 or above. Approximately 25% met DSM-V criteria for BED at baseline. Exclusion criteria included a previous or current regular meditation practice; concurrent participation in a weight loss program; and meeting criteria for purging bulimia, in addition to any indications of active additional need for psychiatric management for depression, drug/alcohol use or other psychiatric disorders, or unstable medication management that might affect weight, eating, or group involvement. In total, 67.5% completed the MB-EAT intervention. There were no differences in demographic characteristics between those who completed the intervention versus those who did not. Imputation of missing values was completed using the last observation carried forward method (Shao and Zhong, 2003).

### Measures

Participants were assessed in a number of areas, including eating and weight related issues, emotional regulation, state mindfulness, and spiritual well-being. Assessment measures reported here include the Binge Eating Scale (BES) (Gormally et al., 1982), the Beck Depression-II and Anxiety Inventories (BDI-II; BAI) (Beck et al., 1996), the Five Facet Mindfulness Questionnaire (FFMQ) (Baer et al., 2008), and Functional Assessment of Chronic Illness Therapy – Spiritual Well-Being Scale (FACIT-Sp) (Peterman et al., 2002).

#### Eating and Emotional Regulation

The BES, rather than being diagnostic of BED, is sensitive to various aspects of compulsive overeating and other eating factors, such as emotional eating and is appropriate for individuals with a wider range of over-eating issues than BED. The measure has good psychometric properties, and in the current study, a Cronbach’s alpha of 0.92 at baseline. The Beck measures of depression and anxiety (BDI-II and BAI) are sensitive to a range of emotional dysregulation at the sub-clinical and clinical levels (current study Cronbach’s alphas: BDI-II = 0.92; BAI = 0.88).

#### Mindfulness

The FFMQ, developed for use with non-meditators and based on factor analysis of a number of previously published measures of mindfulness, has five distinguishable factors: Observe; Describe; Acting with Awareness; Non-judging; and Non-reactive (current study Cronbach’s alphas: Observe = 0.72; Describe = 0.92; Acting with Awareness = 0.86; Non-judging = 0.80; and Non-reactive = 0.54). These entail, respectively, observing present-moment experiences, describing experiences (applying verbal labels), acting with awareness rather than ‘mindlessly’/automatically, accepting present-moment experiences without judgment, and not reacting to negative experiences.

#### Spiritual Well-Being

The FACIT-Sp, containing two factors, Meaning and Peace [Meaning/Peace (M/P)], and Faith, was developed to assess these separable aspects of spiritual/religious involvement in individuals with cancer and other medical challenges. Representative items for Meaning and Peace include: “I am able to reach down deep into myself for comfort”; “I feel a sense of harmony within myself”; and for Faith: “I’ve strengthened my faith or spiritual beliefs.”; “I find comfort in my faith or spiritual beliefs.” This version used contained adapted wording to make it more generally applicable, as used in previous research on mindfulness effects (Carmody et al., 2008) (current study Cronbach’s alphas: M/P = 0.86; Faith = 0.91).

### Procedure

After baseline assessment, eligible participants were randomly assigned within cohorts to the MB-EAT intervention or to a wait list control approximately two weeks prior to the initial treatment session. Participants were provided a brief individual orientation to their assigned condition. Participants were assessed at baseline, midpoint, immediate post, and 1, 2, and 4 months follow-up. Initial post-treatment assessment point was after the 10 weekly sessions. The FACIT-Sp was collected at immediate post and 2 month followup, so these represent the data points reported here.

#### Mindfulness-Based Eating Awareness Training

The MB-EAT program consists of a manualized 12-session intervention (10 weekly sessions with 2 monthly booster sessions). Sessions are 2 aaa hours long, with 2 h for Session 11 and 12. All sessions include both ‘inner’ and ‘outer’ wisdom components. See **Table 1** for core elements. The contrast with traditional, more restrictive approaches to weight management/weight loss is highlighted from the beginning, by focusing on flexibility, making long-term changes, including more ‘indulgent’ foods (though in smaller quantities), and increasing enjoyment of eating. The program continually emphasizes developing sustainable eating patterns in regard to food choice, with an emphasis on day to day flexibility, and developing an attitude of mindful curiosity.

**Table 1:** Outline of sessions for MB-EAT group.

**Session 1:**	Introduction to the Inner Wisdom/Outer Wisdom model; Mindful Raisin Exercise; Introduction to Mindfulness Meditation with practice in group.
**Session 2:**	**Inner Wisdom:** Introduction of ‘mini-meditation’ practice; Mindfully eating challenging food (cheese and crackers). **Outer Wisdom:** Introduction of 500 Calorie Challenge/mindful nutrition/self-tracking.
**Session 3:**	**Inner Wisdom:** Hunger awareness – physical vs. other triggers; Body scan. **Outer Wisdom:** 500 Calorie Challenge – making sustainable changes.
**Session 4:**	**Inner Wisdom:** Taste satisfaction/satiety; Mindfully eating chocolate; Healing self-touch. **Outer Wisdom:** Combining taste awareness with calorie/nutrition awareness.
**Session 5:**	**Inner Wisdom:** Fullness awareness, with water; body satiety; Making mindful food choices (chips vs. cookies); Introducing chain reaction concept; Forgiveness meditation. **Outer Wisdom:** Introduction to awareness of physical activity using pedometers.
**Session 6:**	**Inner Wisdom:** Mindful food choices (fruits and veggies); Integrated mindful eating meditation. **Outer Wisdom:** Nutrition choices/meeting energy needs.
**Session 7:**	**Inner Wisdom/Outer Wisdom:** Pot luck meal. Review of 500 Calorie Challenge.
**Session 8:**	**Inner Wisdom:** Body scan and chair yoga; Eating triggers revisited. **Inner Wisdom/Outer Wisdom:** Mindful walking; increasing physical activity.
**Session 9:**	**Inner Wisdom:** Interrupting the chain reaction; Values exercise; Favorite food exercise. **Outer Wisdom:** Maintaining change in food choices and physical activity.
**Session 10:**	**Inner Wisdom:** Wisdom meditation. **Outer Wisdom:** Healthy snacking.
**Follow-up Sessions 11 and 12:**	Meditation practice; Review of progress; Self-Acceptance meditation; Maintaining and deepening change.

Core forms of meditation include general mindfulness meditation, guided eating meditations, and “mini-meditations” used at meal time and throughout the day. Modeled on the MBSR program (Kabat-Zinn, 1990), general mindfulness meditation first uses the breath as the focus of awareness, followed by open awareness meditation, of whatever thoughts, emotions, or bodily sensations arise. “Mini-meditations,” although developed for this program, conceptually draw on the principle within traditional mindfulness practice of bringing moment-to-moment awareness into all activity. Meditation practice begins with a 10-min general mindfulness meditation, extending to 20-min at Session 3; about half way through the program, individuals are encouraged to try meditating without the audio recording, and to increase their sitting practice to 30 min. See Kristeller and Wolever (2011; Kristeller and Wolever (2011, in press) for an in-depth conceptual overview of program elements.

Multiple guided eating mindfulness practices cultivate awareness of the experiences of physical hunger, taste satisfaction, satiety signals (taste, fullness, and body satiety) and food choice, using small amounts of increasingly challenging foods, in addition to thoughts, feelings and socio-environmental triggers for eating/binging. With the exception of mindfully eating raisins, adapted from the MBSR program, all eating practices were developed for MB-EAT, incorporating foods often identified as frequently over-eaten, including cheese and crackers, chocolate, corn chips and cookies. Healthier foods are also introduced; for a potluck meal in Session 7, participants bring two dishes (one “healthier,” and one less so, representing a food they’d like to continue eating in moderation). This experience integrates all elements of mindful eating within a full meal experience, preparing participants for a culminating home practice assignment of going to an ‘all-you-can-eat buffet.’

Also included throughout the program are scripted guided meditations and other exercises addressing both eating issues and broader life balance issues, related to placing the experience of food, weight concerns, and eating into better balance with other life concerns. These include a forgiveness meditation to address anger-triggered eating, identifying linked triggers leading to overeating, a self-acceptance meditation, and a wisdom meditation. Body awareness practices represent another “inner wisdom” treatment component, including chair-based yoga, a body scan exercise, and healing self-touch, which extends self-acceptance to body size and shape.

As the MB-EAT program developed, “outer wisdom” components, including ways to mindfully address weight management, healthier nutrition, and increase physical activity, have become core components. These include the “500 Calorie Challenge,” eating in response to energy needs, ways to improve nutritional content while retaining favorite foods, and use of a pedometer to raise awareness of and gradually develop physical activity.

Each session begins with brief meditation/body awareness practice, with more extended instruction in the first four sessions. This is followed by 15–20 min for inquiry and discussing progress and difficulties experienced during the previous week. As identified in **Table 1**, each session focuses on a specific theme related to normalizing eating patterns, increasing healthier, more balanced eating, and managing non-nutritional triggers for overeating. Home practices include meditation practice, mindful-eating exercises, and other practices specific to each week’s theme. Individuals who missed a treatment session were offered a brief individual appointment to cover core elements of the missed session, deemed important due to the new components/skills introduced at each session.

#### Wait List Control

The Wait List control participants did not receive treatment during the period in which they were enrolled. Following the final assessment point, they were offered a 4-session workshop version of MB-EAT.

### Statistical Analysis

To test hypothesis 1, that MB-EAT would cultivate spirituality, two-way mixed ANOVAs were conducted to determine the effect of treatment on the FACIT-Sp M/P and Faith measures. To test hypothesis 2, that spirituality would be associated with salubrious emotional and eating behavior outcomes, change scores were calculated for the FACIT factors and BDI-II, BAI, and BES (for both immediate post minus baseline, and 2 month followup minus baseline). Correlation analyses were then used to examine the relationship between change in spirituality and change in emotional and eating behavior outcomes. To test hypothesis 3, that spirituality would be associated with mindfulness, change scores were calculated for the FFMQ factors (immediate post minus baseline), and correlation analyses were conducted to examine the relationship between change in spirituality and change in the FFMQ factors. Finally, to test hypothesis 4, that spirituality would mediate the relationship between changes in mindfulness and emotional and eating behavior outcomes, separate simple mediation models were conducted using a bootstrap estimation approach with the PROCESS macro for SPSS version 24 (Hayes, 2018). All analyses were conducted as intent-to-treat (ITT).

## Results

### Main Effects of MB-EAT on Spirituality

**FIGURE 1:**
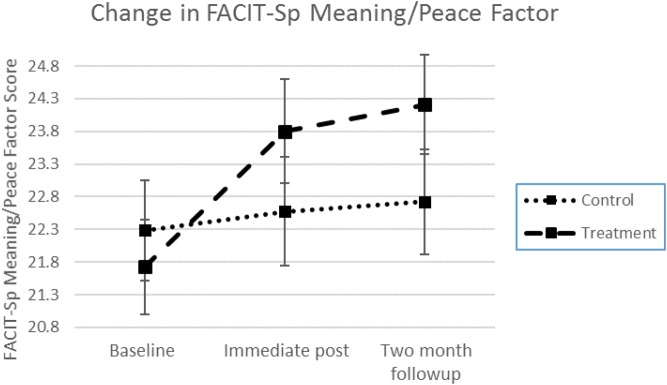
Treatment effects across time for the FACIT-Sp Meaning and Peace factor. Group × time interaction is significant at *p* < 0.01. Error bars represent the standard error of the mean at each time point.

To determine whether the FACIT-Sp Meaning/Peace (M/P) factor changed over the course of treatment and followup, a two-way mixed ANOVA was conducted; see **Table 2** for FACIT-Sp means and standard deviations (along with the means and standard deviations for the other key variables). For the FACIT-Sp M/P factor, there was a statistically significant two-way interaction between treatment and time, *F*(2,228) = 5.03, *p* < 0.01, $\eta_{p}^{2}$ = 0.042, with a medium effect size (IP – Baseline: Cohen’s *d* = 0.45). For the MB-EAT group, scores increased most clearly from baseline to end of treatment, with a small continued increase to 2-month followup remaining generally constant in the control group (see **Figure 1**). Direct comparison of means between the control and treatment groups at baseline, immediate post, and 2 months followup, however, were not significant, though they were in the expected direction [Baseline: *F*(1,114) = 0.28, *p* = 0.60, $\eta_{p}^{2}$ = 0.002; immediate post: *F*(1,115) = 1.14, *p* = 0.29, $\eta_{p}^{2}$ = 0.01; 2 months followup: *F*(1,115) = 1.55, *p* = 0.22, $\eta_{p}^{2}$ = 0.013].

**Table 2:** Means and standard deviations (in parentheses) for the FACIT-Sp factors, BES, BDI, BAI, and FFMQ factors.

	Baseline	Immediate post	Two month followup
	**Control Group (*N* = 56)**		
FACIT-Sp Meaning/Peace	22.28 (6.10)	22.58 (6.23)	22.83 (5.88)
FACIT-Sp Faith	11.13 (4.63)	10.67 (4.96)	10.71 (4.81)
BDI	15.36 (8.57)	11.50 (8.99)	11.14 (8.75)
BES	19.25 (9.39)	17.43 (9.85)	16.32 (9.39)
BAI	29.96 (7.49)	30.05 (7.94)	
FFMQ Observe	25.79 (4.83)	25.97 (5.30)	25.37 (5.76)
FFMQ Describe	27. 31 (6.70)	27.41 (6.98)	27.51 (7.40)
FFMQ Awareness	27.62 (5.52)	27.39 (5.42)	27.42 (5.98)
FFMQ Non-judging	27.66 (5.57)	29.15 (6.18)	29.33 (6.46)
FFMQ Non-reactive	23.15 (4.58)	23.53 (4.92)	23.22 (5.25)
	**Treatment Group (*N* = 61)**		
FACIT-Sp Meaning/Peace	21.73 (5.21)	23.80 (6.11)	24.21 (6.05)
FACIT-Sp Faith	10.37 (4.87)	11.81 (4.23)	11.99 (4.18)
BDI	14.67 (9.36)	8.42 (8.43)	8.25 (9.60)
BES	18.97 (10.12)	12.49 (8.70)	11.83 (9.37)
BAI	29.97 (6.91)	28.95 (7.44)	
FFMQ Observe	27.28 (5.50)	28.28 (4.71)	28.52 (4.95)
FFMQ Describe	27.23 (6.26)	27.62 (6.49)	27.72 (7.18)
FFMQ Awareness	26.69 (5.12)	27.63 (5.64)	28.62 (5.91)
FFMQ Non-judging	26.88 (5.72)	27.48 (6.53)	28.89 (6.53)
FFMQ Non-reactive	21.88 (4.04)	21.76 (3.81)	22.61 (3.54)


**FIGURE 2:**
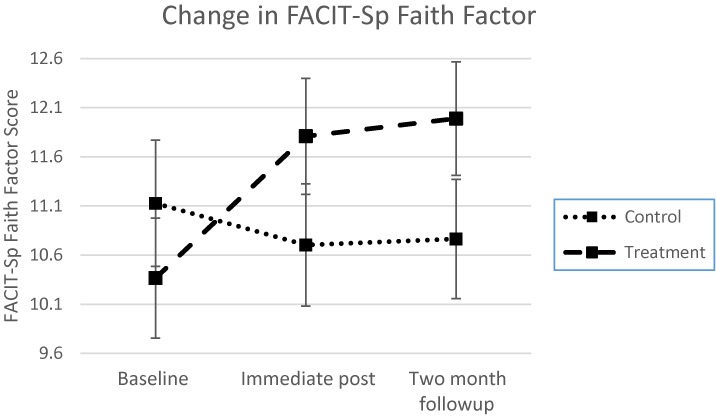
Treatment effects across time for the FACIT-Sp Faith factor. Group × time interaction is significant at *p* < 0.001. Error bars represent the standard error of the mean at each time point.

To determine whether the FACIT-Sp Faith factor changed over the course of treatment and followup, a two-way mixed ANOVA was also conducted. For the FACIT-Sp Faith factor, there was a statistically significant two-way interaction between treatment and time, *F*(2,228) = 13.58, *p* < 0.001, $\eta_{p}^{2}$ = 0.106. In a comparison between the mean change scores (IP minus baseline) for the treatment and control groups, a medium sized effect was demonstrated (Cohen’s *d* = 0.68). The Faith factor increased from baseline to immediate post for the MB-EAT group, decreasing in the control group, and remaining about the same for each group to 2 months (see **Figure 2**). Comparison of means between the control and treatment groups at baseline, immediate post, and 2 months followup, were again not significant though in the expected direction [Baseline: *F*(1,114) = 0.74, *p* = 0.39, $\eta_{p}^{2}$ = 0.006; immediate post: *F*(1,115) = 1.79, *p* = 0.18, $\eta_{p}^{2}$ = 0.015; 2 months followup: *F*(1,115) = 2.35, *p* = 0.13, $\eta_{p}^{2}$ = 0.020].

### Relationships Among Changes in Key Variables

Correlations between change scores between immediate post and baseline were calculated for the FACIT-Sp M/P factor, the FACIT-Sp Faith factor, the BDI-II, the BAI, and the BES. As can be seen in **Table 3**, as M/P and Faith increased, self-reported depressive, anxious, and binge-eating symptoms decreased. These correlations were stronger for the M/P factor of the FACIT-Sp. Correlations between these change scores were also calculated for the FFMQ factors. As can be seen in **Table 4**, change in the Faith factor of the FACIT-Sp was significantly correlated with change in most of the FFMQ factors in the expected direction. For the FACIT-Sp M/P factor, significant correlations were only found with the Describe and Observe factors of the FFMQ.

**Table 3:** Correlation coefficients of change scores (FACIT-Sp factors, BDI, BAI, and BES) for both Immediate Post minus Baseline and 2 Month Followup minus Baseline.

	Immediate Post minus Baseline			Two Month Followup minus Baseline	
	Control Group (*N* = 56)			Control Group (*N* = 56)	
	Meaning/Peace change	Faith change		Meaning/Peace change	Faith change
BDI change	-0.30^∗^	-0.16	BDI change	-0.45^∗∗^	0.06
BAI change	-0.39^∗∗^	-0.05	BAI change		
BES change	0.10	0.23	BES change	-0.13	0.18

	**Treatment Group (*N* = 61)**			**Treatment Group (*N* = 61)**	
	**Meaning/Peace change**	**Faith change**		**Meaning/Peace change**	**Faith change**

BDI change	-0.57^∗∗∗^	-0.30^∗^	BDI change	-0.65^∗∗∗^	-0.27^∗^
BAI change	-0.45^∗∗∗^	-0.36^∗∗^	BAI change		
BES change	-0.45^∗∗∗^	-0.36^∗∗^	BES change	-0.57^∗∗∗^	-0.40^∗∗^

*^∗^*p* < 0.05; ^∗∗^*p* < 0.01; ^∗∗∗^*p* < 0.001. *N* = 56 for control group; *N* = 61 for treatment group.*

**Table 4:** Correlation coefficients of change scores (FACIT-Sp factors and FFMQ factors) for Immediate Post minus Baseline.

	Control Group (*N* = 56)			Treatment Group (*N* = 61)	
	Meaning/Peace change	Faith change		Meaning/Peace change	Faith change
Observe change	-0.27*	0.08	Observe change	0.25*	0.23
Describe change	0.25	0.28*	Describe change	0.33**	0.29*
Awareness change	0.32*	0.07	Awareness change	0.23	0.35**
Non-judging change	0.00	0.15	Non-judging change	0.13	0.34**
Non-reactive change	0.08	0.43**	Non-reactive change	0.20	0.27*

*^∗^*p* < 0.05; ^∗∗^*p* < 0.01. *N* = 56 for control group; *N* = 61 for treatment group.*

To examine whether these positive correlations persisted after treatment, change scores were calculated between the 2 months followup and baseline scores of the FACIT-Sp factors and the BDI-II and BES (the BAI was not completed at 2 months followup). Significant correlations were observed (see **Table 3**) suggesting that increases in M/P and Faith continued to be associated with decreases in depressive and binge eating symptoms 2 months after treatment was completed.

### Mediating Role of Spirituality on Treatment Effects

**FIGURE 3:**
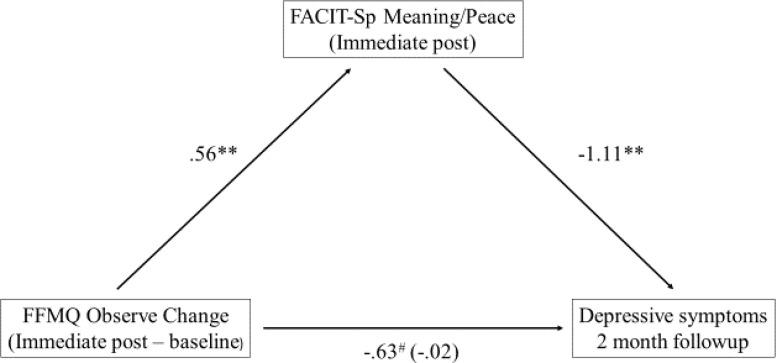
Mediation model between FFMQ Observe and depressive symptoms. ^∗∗^*p* < 0.01; ^#^*p* = 0.05; *N* = 61.

A key exploratory question was whether spiritual well-being played a role in mediating effects of treatment as measured by changes in mindfulness on both emotional and eating regulation. To address this, the PROCESS macro for SPSS version 24 was used (Hayes, 2018) to determine whether the relationship between change in the FFMQ factors (IP minus baseline) and depressive symptoms at 2 months followup was mediated by the FACIT-Sp M/P or Faith factors at the end of treatment. In other words, is there a way in which change in mindfulness (IP – baseline) influences depressive symptoms by way of spiritual well-being? Of the mindfulness factors, initial change in the Observe factor was positively associated with the FACIT-Sp M/P at the end of treatment, and this aspect of spiritual well-being was negatively associated with depressive symptoms at the 2 months followup (see **Figure 3**). The indirect effect was tested with a bootstrap estimation approach (Hayes, 2018). The indirect effect between the Observe factor and depressive symptoms was significant, *b* = -0.62, *SE* = 0.19, 95% CI = -0.983 to -0.260, meaning that a one-point increase in the Observe factor from baseline to IP was associated with approximately a 0.62 point decrease in depressive symptoms as mediated by the FACIT-Sp M/P factor. Of note, the direct effect was no longer significant (*b* = -0.02, *p* = 0.95) once taking account of the mediator, consistent with full mediation. The FACIT-Sp M/P factor did not demonstrate simple mediation between the other FFMQ factors and depressive symptoms. The non-significant indirect effects ranged from -0.17 to 0.24 with 95% confidence intervals overlapping with zero in each instance.

**FIGURE 4:**
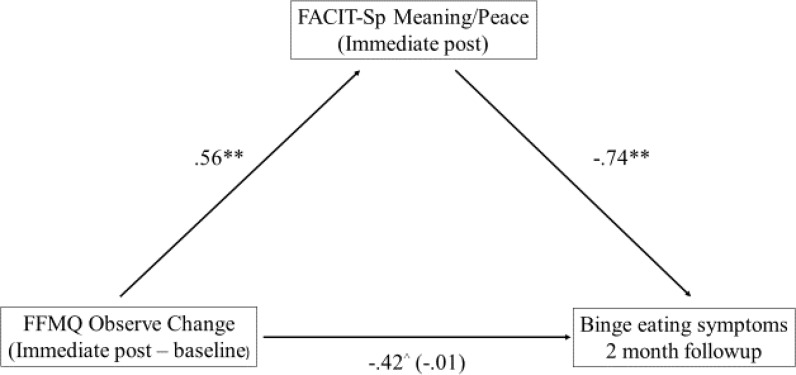
Mediation model between FFMQ Observe and binge eating symptoms. ^∗∗^*p* < 0.01; ^∧^*p* = 0.197; *N* = 61.

Similarly, the question was explored whether change in mindfulness (IP – baseline) influenced binge eating symptoms by way of spiritual well-being? Again, the PROCESS macro for SPSS was used to determine whether changes in the FFMQ factors (IP minus baseline) and binge eating symptoms at 2 months followup were mediated by the FACIT-Sp M/P or Faith at the end of treatment (see **Figure 4**). Similar to the finding with depressive symptoms, the indirect effect between the Observe factor and binge eating symptoms was found to be significant for the FACIT- Sp M/P: *b* = -0.41, *SE* = 0.15, 95% CI = -0.741 to -0.142; again, the direct effect between change in Observe and the BES was not significant (*b* = -0.01, *p* = 0.99) consistent with full mediation. The FACIT-Sp M/P factor did not demonstrate simple mediation between the other FFMQ factors and binge eating symptoms. The non-significant indirect effects ranged from -0.11 to 0.16 with 95% confidence intervals overlapping with zero in each instance.

None of the analyses of the other FFMQ factors, the FACIT-Sp Faith factor, and the BES or BDI-II demonstrated simple mediation. Each simple mediation model yielded non-significant indirect effects ranging from -0.05 to 0.07 with 95% confidence intervals overlapping with zero in each instance.

## Discussion

The results found in this study for the effects of MB-EAT on spiritual well-being and their relationship to other variables are largely parallel to those found in previous research investigating the effects of MBSR on spiritual well-being (Carmody et al., 2008). Although the increases in the MB-EAT program on the FACIT-Sp values from baseline to immediate post were somewhat smaller than for the MBSR program, as is understandable, given the more substantial focus on mindfulness practice *per se* in the MBSR program, they were still meaningful in their relationship to other changes.

Also similar to the results found in the MBSR study are the clear relationships between increases in Meaning/Peace and Faith and decreases in depression anxiety, and program-targeted symptoms: health-related for the MBSR program and binge-eating symptoms for MB-EAT, with these relationships in both studies somewhat stronger for Meaning/Peace than for Faith. In the current study, this pattern was sustained and was even somewhat stronger at the 2-month followup, suggesting the effects of the treatment, particularly via increased Meaning and Peace, were still present past the conclusion of the weekly treatment phase, evidence that was not available for the MBSR study. Overall, the findings related to Meaning and Peace are quite consistent with recent research and writings emphasizing a meaning systems framework for conceptualizing the effects of religiousness and spirituality (Paloutzian, 2017).

The change in the Faith factor was more consistently related to changes in the FFMQ at immediate post, correlating with four factors, whereas change in the Meaning and Peace factor was significantly related only to change on the Describe and Observe factors. This finding is somewhat perplexing because the item content of the Meaning and Peace factor (e.g., purpose, harmony) seems to correspond more fully with the practice of mindfulness. It is possible that the predominantly Christian population in the current study rendered the Faith factor more relevant, especially as it relates to the factors of the FFMQ. At the same time, our exploratory analyses suggest that the Meaning and Peace factor of the FACIT-Sp fully mediates the relationship between initial change (at immediate post) on the FFMQ Observe factor and its relationship with improvement in both uncontrolled/emotional eating and post-treatment depression. One reason the Observe factor may link with the Meaning/Peace factor is that all items loading on this factor are mostly positive in content, related to experiencing body/sensory feelings, such as “I pay attention to sensations, such as the wind in my hair or sun on my face.” The other FFMQ factors are either all or mostly reverse-scored, such that they indicate ‘mindlessness’ as worded, or for ‘Non-reactive,’ tap into NOT reacting to generally negative situations. Future research is needed to examine how engaging mindfulness and other meditation-based interventions relate to different aspects of spirituality.

It is worthwhile to consider further the evidence for the self-regulatory value of spiritual well-being on behavior, consistent with a linkage between spiritual/faith engagement and the concept of wisdom, over and above the positive emotional value of such spiritual experiences or possible effects on depression. Related to this, in our earlier research using MB-EAT with individuals with BED (Kristeller et al., 2013), absolute improvement on the BES and depression was comparable between the two intervention groups. However, for the participants receiving the Psycho-Ed/CBT intervention, improvement in eating regulation was highly correlated to decreases in depression, but completely unrelated to change in depression for those in the MB-EAT condition. Improvement in spiritual well-being, which was not measured in that study, may have therefore been playing a similar important mediating role.

Improvement in compulsive and emotional overeating is largely a function of ongoing behavioral change, suggesting that individuals who experience heightened spiritual well-being, may also be more fully engaging what we refer to as ‘wisdom’ within the intervention, while letting go of the chronic struggles around food, their weight and their sense of self that they had previously experienced. To the degree that this pattern is consistent with mindfulness-based programs, encouraging more active engagement of the spiritual self might be integrated into such programs, consistent with guidelines for doing so (Pargament and Faigin, 2012; Plante, 2016). In addition, it would raise questions about policies that stipulate avoiding doing so as religiously biased or inappropriate. One woman, who entered the program with BED, shared her extended experience with MB-EAT in an informal followup interview several years after completing the program. She noted that she was no longer binging with any frequency, but while she was rarely practicing formal meditation, she frequently used mindfulness and breathe awareness. She also attributed much of her self-growth, not only in regard to eating but also in relation to other areas of her life, to the meditation training and practice, noting “it helps me hook into my inner wisdom. Meditation slows you down enough to be in touch with God … and God lives in all of us” (Kristeller, 2007).

This study has several limitations. First, there was a relatively high drop-out rate, and the possibility of self-selection effects cannot be completely ruled out. However, our drop-out rate is similar to other studies (Carlson et al., 2016). Second, we only used one measure of spirituality. There are other measures available including the Spiritual Transformation Scale (Cole et al., 2008), the Sources of Spirituality Scale (Davis et al., 2015), and the Spiritual Transcendence Scale (Piedmont et al., 2008). Given the debate about the construct of spirituality, using multiple measures, with somewhat distinct conceptualizations of spirituality, may help future researchers to determine which aspects of spirituality are most sensitive to interventions involving mindfulness. Finally, although followup assessment in the current study continued out to 6 months post-intervention, these did not include the FACIT-Sp past 2 months, so that longer term assessment of spiritual engagement might have more fully illuminated the underlying processes. Another limitation is that no measures of wisdom were included; such measures are now under further development (Jeste et al., 2010; Noghabi and Nabizadeh, 2017).

The psychology of spirituality continues to move in exciting directions and the rich history of mindfulness and associated interventions will continue to cross paths with this field. This is a good thing. As a universal human capacity, understanding the psychology of spirituality needs to be engaged rather than ignored, neglected, or written off. In the quickened world we live in, slowing down the associated reactivity is difficult, but to the extent that mindfulness can do this, the human capacity of spirituality – which has served humanity for millennia – can continue to do so in the 21st century.

## Author’s Note

Further information regarding the MB-EAT program used here may be obtained from JK, at Jean.Kristeller@indstate.edu. A published version is in press.

## Ethics Statement

This study and associated protocol were approved by the Institutional Review Board at Indiana State University. All subjects gave written informed consent in accordance with the Declaration of Helsinki.

## Author Contributions

JK created the MB-EAT program and was the principal investigator for the current study. JK and KJ contributed to the introduction, materials and methods, results, and discussion sections. KJ has collaborated with JK on research related to spirituality, mindfulness, and health.

## Conflict of Interest Statement

The authors declare that the research was conducted in the absence of any commercial or financial relationships that could be construed as a potential conflict of interest.
